# Transcriptome Analysis and Reactive Oxygen Species Detection Suggest Contrasting Molecular Mechanisms in *Populus canadensis*’ Response to Different Formae Speciales of *Marssonina brunnea*

**DOI:** 10.3390/genes15010116

**Published:** 2024-01-18

**Authors:** Yanfeng Zhang, Longyan Tian

**Affiliations:** 1 School of Ecological Engineering, Guangdong Eco-Engineering Polytechnic, Guangzhou 510520, China; 2 Guangdong Provincial Key Laboratory of Silviculture, Protection and Utilization, Guangdong Academy of Forestry, Guangzhou 510520, China

**Keywords:** differentially expressed genes (DEGs), plant–pathogen interaction, poplar, resistance

## Abstract

Revealing plant–pathogen interactions is important for resistance breeding, but it remains a complex process that presents many challenges. *Marssonina* leaf spot of poplars (MLSP) is the main disease in poplars; in China, its pathogens consist of two formae speciales, namely, *Marssonina brunnea* f. sp. *Monogermtubi* (MO) and *M. brunnea* f. sp. *Multigermtubi* (MU). However, the mechanism of the molecular interaction between poplars and the two formae speciales, especially for an incompatible system, remains unclear. In this study, we conducted transcriptome sequencing and reactive oxygen species (ROS) staining based on the interactions between *Populus canadensis* and the two formae speciales. The results show that the gene expression patterns of *P. canadensis* induced by MO and MU were significantly different, especially for the genes associated with biotic stress. Furthermore, MO and MU also triggered distinct ROS reactions of *P. canadensis*, and ROS (mainly H_2_O_2_) burst was only observed around the cells penetrated by MU. In conclusion, this study suggested that *P. canadensis* experienced different resistance reactions in response to the two formae speciales of *M. brunnea*, providing valuable insights for further understanding the host–pathogen interactions of MLSP.

## 1. Introduction

Revealing plant–pathogen interaction is crucial for improving resistance breeding; however, it is largely a complex process, involving compatible and incompatible reactions and presenting many challenges [1,2]. For fungal pathogens, individual strains of some species, like *Fusarium oxysporum* and *Puccinia graminis*, are host-specific; in other words, they are formae speciales [3,4]. Generally, different formae speciales have similar morphological features, but have independent host ranges and, on the whole, cannot infect the hosts of other formae speciales [4,5]. Apparently, there are underlying defense mechanisms in the interactions between formae speciales and their hosts. In fact, a few researchers have focused on this point and found that different formae speciales mostly display gene-for-gene relationships and establish specific interactions with their corresponding host; furthermore, some biological processes, involving oxidative burst, enzymatic hydrolysis, cell wall dynamics, etc., have been proven to participate in the building of these interaction systems, but the definite mechanism remains unclear [6,7,8,9]. In addition, researchers also found that pathogens that contain different formae speciales are mostly highly adaptable and variable in virulence [10,11], which brings many additional barriers in terms of durable resistance. Therefore, clarifying the molecular mechanism between formae speciales and their hosts is necessary for sustainable breeding in the future.

MLSP, as a serious worldwide disease in poplar plantations, is mainly caused by *M. brunnea*. In China, *M. brunnea* has been reported to contain two formae speciales, namely, MO and MU; under natural conditions, the former mainly infects poplar hosts from *Populus* sect. *Leuce*, while the latter specifically infects poplar hosts from *Populus* sect. *Aigeiros* [12,13]. *M. brunnea* can been used as a model pathogen in woody plants for studying a forma specialis because of its stable interaction, easy experimental manipulation, and sufficient research bases (involving a clear infection process, whole genome data, and pathogenic gene analyses) [14,15,16,17]. In fact, a few studies have analyzed the interactions between *M. brunnea* and its poplar host. Through transcriptome sequencing, the gene expression patterns associated with different interaction systems, involving MU with susceptible and resistant *Aigeiros* hosts and MO with a susceptible *Leuce* host, were clearly revealed [18,19,20,21]. Using multigene association studies and physiological experiments, photosynthesis was proven to be closely related to the MLSP disease process [22,23]. Moreover, the molecular basis of the poplar immune system against MU was also partially discovered [24,25,26,27]. These researchers have provided important evidence in explaining the pathogenic process and host defense mechanism of *M. brunnea*, but their studies have mainly been focused on MU or compatible interactions. Thus, questions like “what reactions happen in *Populus* sect. *Aigeiros* in response to MO?” and “how do poplar species interact with these two formae speciales?” remain unanswered.

In conclusion, as studies on incompatible interactions between poplars and the two formae speciales of *M. brunnea* are lacking, conducting relevant research might bring new knowledge for MLSP; furthermore, based on formae speciales research into other fungal pathogens, immunity responses might also participate in establishing the interaction systems of MLSP. To further explore the potential molecular mechanism of poplars in response to *M. brunnea*, we compared the gene transcription and ROS release of *P. canadensis* leaves in response to MO (incompatible) and MU (compatible).

## 2. Materials and Methods

### 2.1. Host and Pathogen

The cultivar of poplar used was *P. canadensis*; cuttings were obtained from the campus of China Three Gorges University, Yichang City, Hubei Province, China (30°43′38.99″ N, 111°18′36.04″ E). The poplar cuttings were cultured in a greenhouse maintained at 25 ± 2 °C under a relative humidity (RH) of 50–55% during the day, and at 22 ± 2 °C under 55–65% RH at night. Light during the day (from 8:00 to 20:00) was supplied using full-spectrum lamps. 

The studied strains were MO1 (*M. brunnea* f. sp. *Monogermtubi*, isolated from *P. tomentosa* in Beijing, China; 40°01′00.23″ N, 116°23′23.50″ E) and MU1 (*M. brunnea* f. sp. *Multigermtubi*, isolated from *P. canadensis* in Beijing, China; 40°00′16.12″ N, 116°20′34.07″ E). For simplicity, as no other strain was used in this study, we marked the two strains with “MO” and “MU”, respectively. In this experiment, the strains were cultured on PDA (potato dextrose agar) plates at 25 °C in the dark using a constant temperature incubator. The plates were collected at 15 days post inoculation to observe the colony morphology.

### 2.2. Inoculation, Microscopy, and Sample Collection

The inoculation and microscope observation were performed following the procedures previously described [14]. The spore suspension (approximately 5 × 10^5^ conidia/mL) was obtained by washing conidia from incubated leaves using sterile water. Fully expanded healthy leaves were collected and placed on wet filter paper (Cytiva, Hangzhou, China) in Petri dishes (Beijing Labgic Technology, Beijing, China) for subsequent inoculation. The spore suspension was sprayed onto the surface of the leaves and the Petri dishes containing inoculated leaves were incubated at 25 °C under a 12 h photoperiod using full-spectrum lamps. For observation, the inoculated leaves were cut into 1 × 1 cm pieces. After decoloring using saturated trichloroacetic acid bleaching liquid and straining with saturated chloral hydrate-aniline blue mixed stain solution, the samples were observed under a light microscope. 

The MO, MU, and control group underwent the same inoculation procedure and were collected at the same time after being inoculated; the experiments were performed in three replicates. For control purposes, the leaves were sprayed with distilled water. Finally, the samples were frozen in liquid nitrogen and stored at −80 °C for RNA isolation. 

### 2.3. RNA Isolation and Sequencing

The RNA extraction of the samples was performed using TRIzol reagent (Invitrogen, Carlsbad, CA, USA) according to the instruction manual. The quality of all RNA was confirmed through agarose gel electrophoresis and determined with a NanoDrop ND-1000 spectrophotometer (Wilmington, DE, USA) and Agilent Bioanalyzer 2100 (Agilent, Santa Clara, CA, USA).

For library construction, 1 µg total RNA per sample was used as input material for the RNA sample preparations. The complementary DNA (cDNA) library was generated using NEBNext^®^ Ultra TM RNA Library Prep Kit for Illumina^®^ CatalogE7530L following the manufacturer’s recommendations. Briefly, the enriched mRNA was purified from total RNA using oligo d(T)25 magnetic beads. Fragmentation was carried out using divalent cations under elevated temperatures in NEBNext First Strand Synthesis Reaction Buffer (5X). After the first-strand cDNA and the second-strand cDNA were synthesized, the selection of purified double-stranded cDNA was performed with AMPureXP beads (Beckman Coulter, Beverly, MA, USA) about 250 bp in size. Then, the library quality was assessed on the Agilent Bioanalyzer 2100 system. Finally, the constructed cDNA libraries were sequenced on a flow cell using an Illumina NovaSeq TM 6000 platform (Illumina; San Diego, CA, USA). The RNA-seq data are available from the National Center for Biotechnology Information (NCBI) database with the BioProject ID: PRJNA1042802.

### 2.4. Read Mapping

*Populus trichocarpa* was selected as the reference species for read mapping. The genome files were downloaded from the JGI database (https://phytozome-next.jgi.doe.gov/info/Ptrichocarpa_v4_1; accessed on 5 October 2022). An index of the reference genome was built using Hisat2 v2.0.5 and paired-end clean reads were aligned to the reference genome using Hisat2 v2.0.5. We selected Hisat2 as the mapping tool as it can generate a database of splice junctions based on the gene model annotation file and, thus, a better mapping result than other non-splice mapping tools.

### 2.5. DEG Analysis and Gene Annotation

DEGs were analyzed using the DESeq2 R package [28] with an identity standard of a false discovery rate (FDR) cut-off of 0.05 and log2 (fold change) ≥ 2. The identification of unique or overlapping DEGs within the samples was performed using Draw Venn Diagram (https://bioinformatics.psb.ugent.be/webtools/Venn/). 

Gene Ontology (GO) enrichment and Encyclopedia of Genes and Genomes (KEGG) enrichment were performed using agriGO v2.0 [29] and the clusterProfiler R package [30]. Additionally, the gene enrichment of DEGs in the biotic stress pathway was visualized using MapMan v3.5.1R2 [31].

### 2.6. Reverse-Transcription (qRT)-PCR

RNA samples were firstly purified with DNase I (RNase-free) (Takara Biomedical Technology (Beijing), Beijing, China). qRT-PCR was performed with Oligo-DT and SuperScript III reverse transcriptase (Invitrogen). All qRT-PCR reactions were performed with SuperReal Premix Plus (SYBR green kits; TIANGEN, Beijing, China) and carried out on an ABI 7500 real-time PCR system (Applied Biosystems, Waltham, MA, USA). Relative expression levels were calculated using the ΔΔCT method, with 60S used as the internal control. All primers used in this study are listed in Supplementary Table S1.

### 2.7. Nitroblue Tetrazolium (NBT) and 3,3-Diaminobenzidine (DAB) Staining

NBT and DAB staining were used for detecting the generation of O_2_^−^ and H_2_O_2_ from *P. canadensis* leaves inoculated with MO and MU. 

For NBT staining, the inoculated leaves were firstly put into a centrifuge tube containing 50 mL NBT solution (0.1%, pH = 7.8) (Coolaber, Beijing, China). After vacuum extraction for about 20–30 min under −0.1 MPa, the samples were incubated in NBT solution and kept for 60 min at room temperature. Then, 1 cm segments cut from the inoculated leaves were decolored twice with 95% (*v*/*v*) ethanol for about 10 min before microscopic examination.

For DAB staining, the inoculated leaves were immersed in 1 mg/mL DAB (Coolaber, Beijing, China) with vacuum extraction for 20–30 min under −0.1 MPa. The samples were incubated in DAB solution at room temperature overnight. Then, 1 cm segments cut from the inoculated leaves were decolored with 95% (*v*/*v*) ethanol at 80 °C until they were clear for microscopic examination.

## 3. Results

### 3.1. Construction of the Interaction System and Formation of Penetration Structure

**Figure 1:**
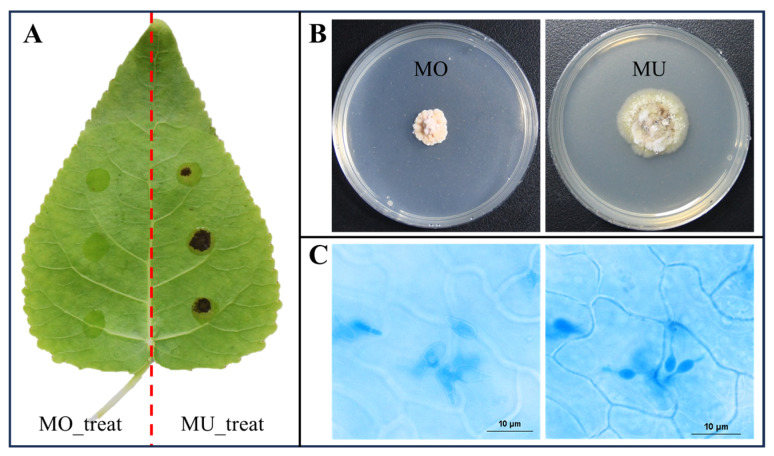
Poplar leaves infected by *M. brunnea*, the cultural colonies, and the penetration process of MLSP. (**A**) At 7 dpi, no visible spots occur at the point inoculated with the spore suspension of MO, while obvious black spots occur at the point inoculated with MU on the leaf of *P. canadensis*. (**B**) The fungal colony of MO is magenta on PDA and the fungal colony of MU is greenish on PDA. (**C**) The left picture shows spores of MU on the surface of the poplar leaf at 8 hpi; the right picture shows the IVs formed in epidermis cells under the spores shown in the left picture.

The inoculation result of 7 dpi (days post inoculation) showed that the leaf of *P. canadensis* was susceptible to MU and resistant to MO (Figure 1A). To further confirm the forma specialis type of the two isolates, we compared their cultural characteristics; the results showed that MO and MU formed magenta and greenish colonies on the PDA culture media, respectively (Figure 1B). 

A histopathological observation of the compatible interaction (*P. canadensis*-MU) showed that MU had penetrated into the host cuticle and formed infection vesicles (IV) in epidermis cells at 8 hpi (hours post inoculation) (Figure 1C), indicating that the parasitic relationship had been established.

### 3.2. Summary of RNA Sequencing

According to the results of the histopathological observation, we chose samples at 8 hpi (an initial infection phase) for transcriptional analysis. 

In total, about 28.0 GB of clean data were obtained in this study. The results show that the Q20/30 values of the detected samples were all over 90% and the mapped rates were all over 75% (76.22–97.4%) (Table 1). Furthermore, duplicate samples from the same treatment can cluster together in PCA and heatmap analyses (based on the expression level of all genes) (Supplementary Figure S1), indicating high-quality sample repeatability. To validate the expression patterns identified using RNA-Seq, eight common genes (Supplementary Table S1) with different expression levels were selected for qRT-PCR analysis. An analysis of the RNA-Seq and qRT-PCR datasets substantiated the expression results generated using RNA-Seq (Supplementary Figure S2).

### 3.3. Analysis of Differentially Expressed Genes (DEGs)

**Figure 2:**
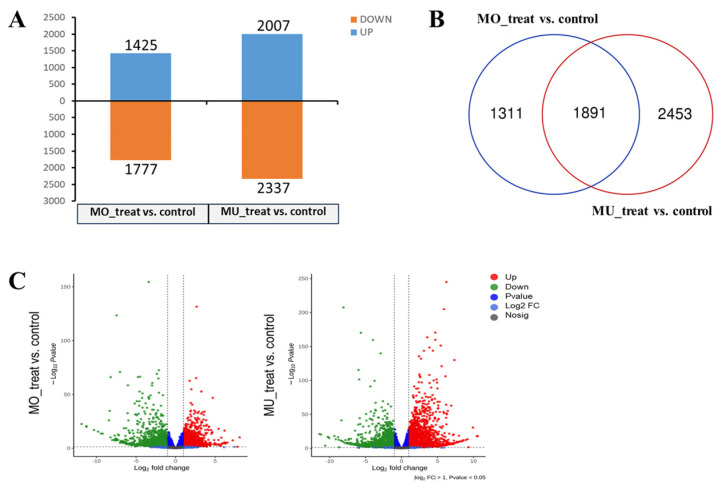
A common comparative analysis of poplar DEGs between MO_treat and MU_treat. (**A**) The number of up− and down−regulated DEGs shown with a histogram. (**B**) Venn diagram showing the number of overlapping genes between the two treatments. (**C**) Volcano plot showing the distribution and significance of DEGs.

According to the statistical results, 3202 (1425 up-regulated, 1777 down-regulated) and 4344 (2007 up-regulated, 2337 down-regulated) DEGs of *P. canadensis* were identified in response to MO and MU infection, respectively (Figure 2A). The Venn diagram shows that 1891 DEGs were shared by the two samples; meanwhile, 1311 DEGs were specific to MO_treat and 2453 DEGs were specific to MU_treat (Figure 2B). The volcano plot shows the expression levels of DEGs. The results indicate that it was easy to find that DEGs of MU_treat presented a wider distribution of expression levels than those of MO_treat (Figure 2C). As a summary, the general comparative analyses of DEGs suggested that *P. canadensis* produced different reactions in response to MO and MU infection. 

### 3.4. GO and KEGG Enrichment Analysis

Both obvious differences and several similarities were found between MO_treat and MU_treat in the GO and KEGG enrichment analyses.

**Figure 3:**
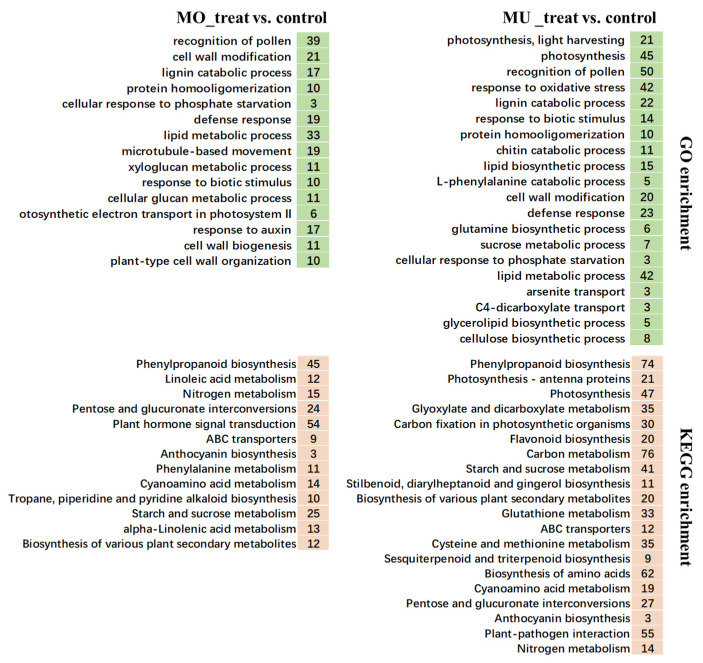
The enriched GO and KEGG terms analyzed by the DEGs of MO_treat vs. control and MU_treat vs. control. In this figure, only the GO terms belonging to biological processes are listed with a selection condition of *p* value < 0.01. The KEGG terms shown in this figure were also selected with a *p* value < 0.01. The values shown in color represent the gene number enriched in each term.

The results of the GO enrichment analysis show that, among the biological processes enriched by the DEGs of MO_treat vs. control, the most significant term was GO:0048544 (recognition of pollen); furthermore, the terms associated with plant defense, involving GO:0042545 (cell wall modification), GO:0006952 (defense response), GO:0010411 (xyloglucan metabolic process), and GO:0009607 (response to biotic stimulus), were also enriched (Figure 3 and Supplementary Table S2). For MU_treat, GO:0009765 (photosynthesis, light harvesting) and GO:0015979 (photosynthesis) were the most significant enriched terms (Figure 3). Similar to MO_treat, GO:0048544 (recognition of pollen), GO:0046274 (lignin catabolic process), and GO:0009607 (response to biotic stimulus) were also enriched; however, the other Go terms related to plant disease resistance, like GO:0006979 (response to oxidative stress), GO:0006032 (chitin catabolic process), GO:0008610 (lipid biosynthetic process), and GO:0006559 (L-phenylalanine catabolic process), were specific to MU_treat (Figure 3 and Supplementary Table S2).

In the KEGG analysis, the numbers of terms significantly enriched by the DEGs from MO_treat vs. control and MU_treat vs. control were 23 and 35, respectively. Among them, a total of 17 terms, including ko00500 (starch and sucrose metabolism), ko00940 (phenylpropanoid biosynthesis), and ko00941 (flavonoid biosynthesis), were shared by MO_treat and MU_treat; meanwhile, “phenylpropanoid biosynthesis” was the most significantly enriched term for both treats (Figure 3 and Supplementary Table S3). Apparently, MU_treat saw a much more complex response, because more terms were enriched and about half of the terms were different in comparison to MO_treat. In the specific terms of MU_treat, ko00195 (Photosynthesis) showed a higher significance; furthermore, it is worth mentioning that a total of 55 genes (encoded proteins including 10 3-ketoacyl-CoA synthases, 10 calcium-binding proteins CML, 9 disease resistance proteins RPM1, 6 respiratory burst oxidases, 6 calcium-dependent protein kinases, 3 pathogenesis-related proteins 1, 2 WRKY transcription factors, 1 mitogen-activated protein kinase, etc.) were enriched in ko04626 (plant-pathogen interaction) (Figure 3, Supplementary Figure S3 and Supplementary Table S3). 

### 3.5. Analysis of Biotic Stress Response Differentially Expressed Genes

Mapman version 3.7.0 was utilized for analyzing the host biotic stress response (plant–pathogen interaction pathways) based on DEGs from MO_treat vs. MU_treat. 

**Figure 4:**
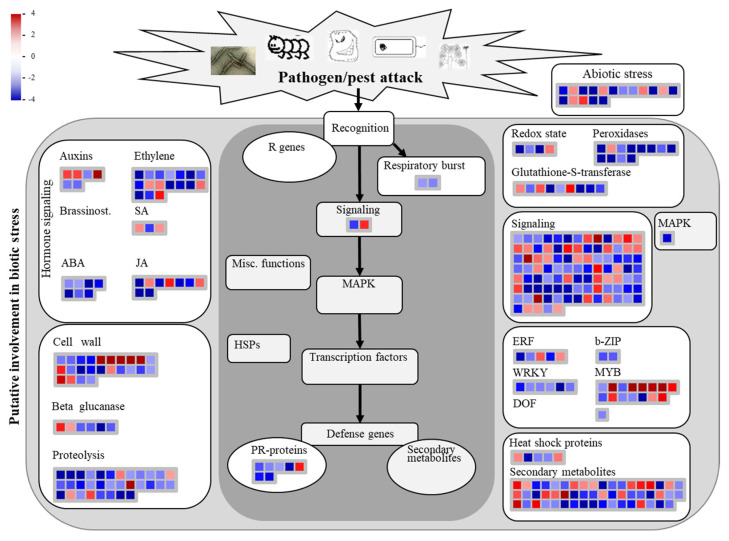
MapMan analysis illustrating the DEGs of MO_treat vs. MU_treat involved in plant–pathogen interaction pathways. The scale showing the value of log2(fold change). The gene details are listed in Supplementary Table S4.

The results showed that over half of the DEGs involved in these pathways were down-regulated, especially for terms like “Ethylene”, “ABA”, “JA”, “Cell wall”, “Proteolysis”, “Respiratory burst”, “PR-proteins”, “Peroxidases”, “b-ZIP”, and “WRKY” (Figure 4 and Supplementary Table S4). Furthermore, for MU_treat, the expression levels of most DEGs involved in “Peroxidases” were apparently higher compared to the control and MO_treat (Supplementary Figure S4). It is remarkable that, in some terms, like “Auxins”, “JA”, and “MYB”, the involved DEGs were mostly up-regulated (Figure 4 and Supplementary Table S4).

### 3.6. Oxidative Burst

Based on the results of the GO and KEGG enrichment, we speculated that the levels of ROS produced by *P. canadensis* would display different responses to the two formae speciales in the conducted detection analysis. NBT and DAB staining were used for detecting the induction of O_2_^−^ and H_2_O_2_ by *M. brunnea* infection in the *P. canadensis* leaves, respectively. 

**Figure 5:**
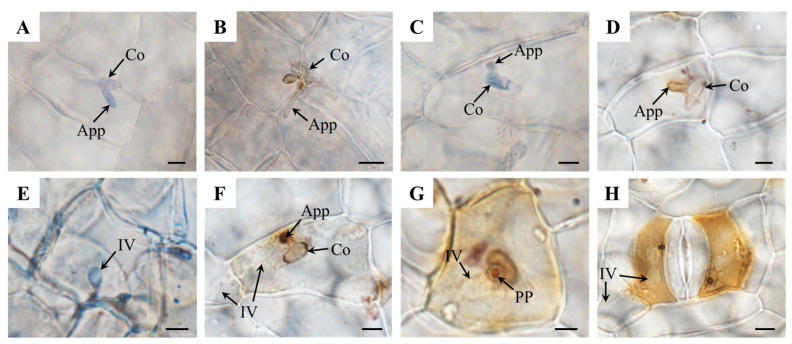
Cytochemical localization of H_2_O_2_ and O_2_^−^ production in poplar–*M. brunnea* interactions. (**A**) An App produced by a Co of MO, stained with NBT. (**B**) An App produced by a Co of MO, stained with DAB. (**C**) An App produced by a Co of MU, stained with NBT. (**D**) An App produced by a Co of MU, stained with DAB. (**E**) IVs formed in the host epidemic cells, stained with DAB. (**F**) An App germinated by a Co of MU on the surface of the poplar upper epidermis and IV formed in the epidemic cells under the Co, stained with DAB. (**G**) A penetration pore (PP) produced by MU on the host surface and IV formed under PP, stained with DAB. (**H**) IV of MU formed in the cells surrounding stomatal guard cells, stained with DAB. Bar = 5 μm.

The conidium (Co) of MO could germinate and form an appressorium (App) on the surface of *P. canadensis*, but could not penetrate into the epidemic cells; the staining results show that both O_2_^−^ and H_2_O_2_ could be detected in the Co and the App, but obvious accumulations of O_2_^−^ and H_2_O_2_ were not found in the host cells under the Co (Figure 5A,B). 

For MU, the distribution of O_2_^−^ and H_2_O_2_ around conidia and appressoria was similar with MO, and there was no oxidative burst around the uninfected cells at the inoculation sites either, but O_2_^−^ and H_2_O_2_ tended to accumulate surrounding the tip of the App compared to MO (Figure 5C,D). In the host epidemic cells that were infected, some O_2_^−^ was detected, mainly germinated around IV (Figure 5E). At the same time, significant H_2_O_2_ aggregation gradually occurred at the penetration sites, especially at the end of the App and surrounding the penetration pore (Figure 5F,G). As the infection structure developed, the burst of H_2_O_2_, with a certain scale, firstly occurred in the host’s infected cells and mostly coincided with the beginning of IV formation; however, there was largely no ROS (reactive oxygen species) burst in the initially penetrated cells and the unpenetrated neighboring cells (Figure 5F–H). 

## 4. Discussion

In recent years, although the mechanism of *M. brunnea*–poplar interaction has gradually gained attention, and although some comprehensive gene expression analyses have been performed, studying incompatible interactions might be much more meaningful for poplar resistance breeding; however, previous studies have mainly been focused on MU–poplar interactions or compatible MO–poplar interactions and limited attention has been paid to the incompatible interactions between the two formae speciales and poplars [15,18,19,21,23]. In this study, we compared the gene expression patterns and the ROS production of *P. canadensis* in response to MO and MU infection, firstly exploring the molecular mechanism of interactions between poplars and the incompatible forma specialis of *M. brunena*. As a result, our study identified the crucial genes associated with plant–pathogen interactions in MLSP and suggested that ROS play an important role in the infection strategies of *M. brunnea*.

Studying plant–pathogen interactions of different fungal formae speciales is crucial for controlling plant pathogens [8,32]. Transcriptome analysis, as an effective method for understanding plant–pathogen interactions, has provided many valuable results; when comparing compatible and incompatible plant–pathogen interactions, differences are mainly found in aspects like transcription factors, hormone signaling, cell wall, heat shock proteins, pathogen-related proteins, etc. [33,34,35]. Similarly, the comparison analysis between MO_treat and MU_treat in this study showed that the differences were distributed in most aspects mentioned here and that many genes associated with cell wall, peroxidases, MYB transcription factors, and secondary metabolites were significantly differently expressed (Figure 3 and Figure 4). For achieving effective resistance, plants could trigger several defense responses through recognizing conserved microbial structures and pathogen virulence molecules [36]. In the GO analysis, the DEGs of MO_treat vs. control and MU_treat vs. control were enriched in terms like “defense response” and “response to biotic stimulus”, respectively (Figure 3), suggesting that *P. canadensis* might successfully recognize MO and MU. Furthermore, for MU_treat, the genes encoding calcium-dependent protein kinases (CDPKs) and respiratory burst oxidase homologues (RBOHs) were mostly up-regulated (Supplementary Figure S3). As reported, CDPKs and RBOHs were both central factors for plants in triggering ROS release in biotic stress responses and were mainly regulated through calcium signaling [37,38], so we speculated that *P. canadensis* might proceed along a similar pathway to produce ROS in response to MU penetration. 

In fact, obvious differences were proven to exist between the compatible interactions caused by MO and MU. These differences relate to several aspects, like cell wall metabolism and phenylpropanoid metabolism [18]. Furthermore, though incompatible interactions have not been analyzed, researchers have previously compared the transcriptomes of *Aigeiros* poplars (clone NL895) infected by highly and weakly active strains of *M. brunnea* f. sp. *multigermtubi*, and found that the DEGs were apparently enriched in GO terms associated with “plant cell death”, which have been proven to be associated with plant disease resistance or susceptibility [19,39]. However, when analyzing MO_treat and MU_treat in our study, the poplar DEGs could be associated with biological processes like “response to biotic stimulus” and “response to oxidative stress” (Supplementary Figure S5), suggesting that the disease resistance of poplar leaves to *M. brunnea* is different between inter- and intra-formae speciales. 

ROS are essential for many botanic biological processes. H_2_O_2_ and O_2_^−^, as the main species of ROS, could directly harm the pathogen body and induce hypersensitive reactions or systemic-acquired resistance; therefore, they act as crucial participators in plant defense mechanisms [40]. In this study, we did not detect any obvious ROS burst in the incompatible MO–poplar interaction; however, when we analyzed the compatible MU–poplar interaction, an obvious H_2_O_2_ release was observed in the infected cells and this was mainly located around IVs and the penetrated sites. So, the ROS production differences of poplar between MO_treat and MU_treat were clear. For other fungal pathogens like *Puccinia striiformis*, a biotrophic pathogen, the attacked host cells could also release an amount of ROS molecules during the infection’s progression and they mostly coincided with the beginning of haustorium formation [41]. For MU, the induced ROS reaction is similar to *P. striiformis*. It had been proven that ROS-induced plant defenses mostly increase resistance to biotrophic pathogens; meanwhile, an excessive production of ROS could also cause plant oxidative stress and benefit the disease development of necrotrophic pathogens [42]. Therefore, considering that *M. brunnea*, as a hemibiotroph, undergoes a biotrophic phase and a necrotrophic phase during the infection process [14], ROS might have both of the above impacts in the development of MLSP. Furthermore, peroxidases were proved to be necessary for controlling ROS levels and prevent their toxicity to plants [43]. In this study, although the peroxidase genes, including seven peroxidase X genes and five anionic peroxidase genes, were mostly up-regulated in MU_treat, this did not solve the problem of ROS burst, indicating that *M. brunnea* might have interfered in the ROS detoxification process of poplars during the MU–*P. canadensis* interaction. As ROS play important roles in the modulation of cell survival, cell death, and biotic stress for plants, further studies on its functions in poplar–*M. brunnea* interactions should be carried out.

## 5. Conclusions

In conclusion, this study firstly compared the host gene transcriptions of the compatible and incompatible interactions between poplars and different formae speciales of *M. brunena*. The results suggested that *P. canadensis* engendered different responses to MO and MU. Specifically, both MO and MU caused the host to respond to the biotic stimulus, but they might trigger different gene expression patterns in reactions like cell wall modification and ROS metabolism. Furthermore, the disease resistance of poplar leaves to *M. brunnea* should be different between inter- and intra-formae speciales. Moreover, staining analysis proved that the ROS production of *P. canadensis* was apparently different between MO_treat and MU_treat. ROS release is important in establishing the compatible interaction system of *P. canadensis*–*M. brunnea*, but is not crucial for incompatible interactions. These results are valuable for further understanding the host–pathogen interaction of MLSP and explaining the pathogenic difference in fungal formae speciales.

## Figures and Tables

**Table 1:** Statistics of reads mapped onto the poplar reference genomes.

SampleID	Clean Reads	Q20	Q30	Input Reads	Mapped Reads	Mapped Rate
control_8hpi_1	41,519,136	97.83%	93.10%	41,519,136	35,888,940	86.44%
control_8hpi_2	38,736,712	97.86%	93.15%	38,736,712	33,457,893	86.37%
control_8hpi_3	37,580,346	97.75%	92.78%	37,580,346	32,366,453	86.13%
MO_8hpi_1	43,733,376	97.55%	92.37%	43,733,376	37,744,736	86.31%
MO_8hpi_2	44,294,756	97.58%	92.47%	44,294,756	38,352,233	86.58%
MO_8hpi_3	38,157,696	98.12%	93.76%	38,157,696	33,351,061	87.40%
MU_8hpi_1	43,474,240	97.68%	92.84%	43,474,240	33,134,118	76.22%
MU_8hpi_2	47,154,366	97.60%	92.63%	47,154,366	36,744,352	77.92%
MU_8hpi_3	42,583,954	97.68%	92.82%	42,583,954	36,758,674	86.32%

## Data Availability

The datasets used and/or analyzed during the current study are available from the corresponding author on reasonable request.

## Supplementary Materials

The following supporting information can be downloaded at: https://www.mdpi.com/article/10.3390/genes15010116/s1, Supplementary Table S1. qRT-PCR primers used in this study; Supplementary Table S2. Enriched terms in the GO analyses based on the DEGs of MO_treat vs. control and MU_treat vs. control (*p* < 0.05); Supplementary Table S3. Enriched terms in the KEGG analyses based on the DEGs of MO_treat vs. control and MU_treat vs. control (*p* < 0.05); Supplementary Table S4. Details of Mapman analysis; Supplementary Figure S1. Correlation among RNA-seq samples; Supplementary Figure S2. Comparison of expression values measured using qRT-PCR and RNA-seq.; Supplementary Figure S3. Annotation and expression profiles of genes involved in plant–pathogen interaction (ko04626) enriched by the DEGs of MU_treat vs. control; Supplementary Figure S4. The expression level of genes belonging to the module “Peroxidases” in the Mapman analysis; Supplementary Figure S5. The enriched GO terms (biological process) analyzed by the DEGs of MO_treat vs. MU_treat and NL895-H vs. NL895-W.
