# Supplementary material for: Transcriptome Analysis and Reactive Oxygen Species Detection Suggest Contrasting Molecular Mechanisms in Populus canadensis’ Response to Different Formae Speciales of Marssonina brunnea
Source: Genes (Basel). 2024 Jan 18;15(1):116. doi: 10.3390/genes15010116 (PMC10815367; doi:10.3390/genes15010116)

Supplementary Figure S1. Correlation among RNA-seq samples. (**A**) heat map of the Pearson correlation of sequenced samples based on the expression data of all genes. (**B**) principal component analysis (PCA) of RNA-seq samples based on the expression data of all genes.

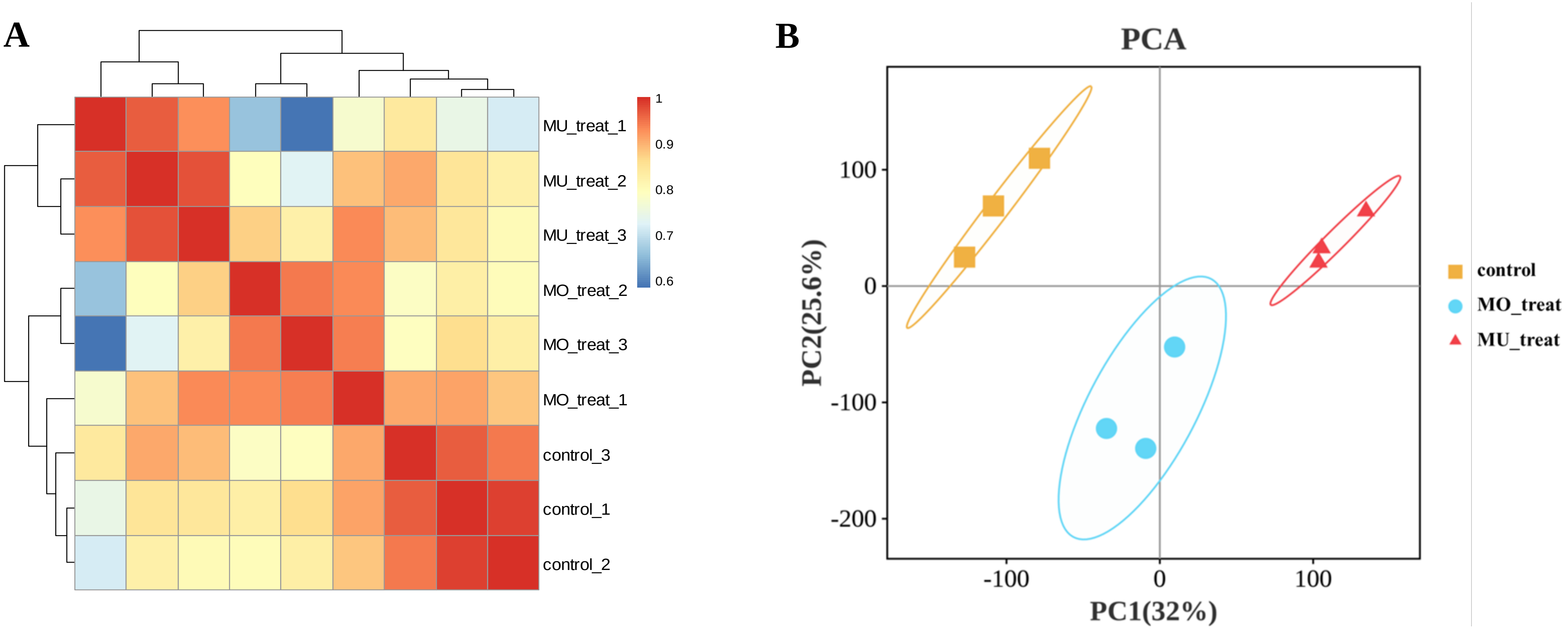

Supplementary Figure S2. Comparison of expression values measured by qRT-PCR and RNA-seq.

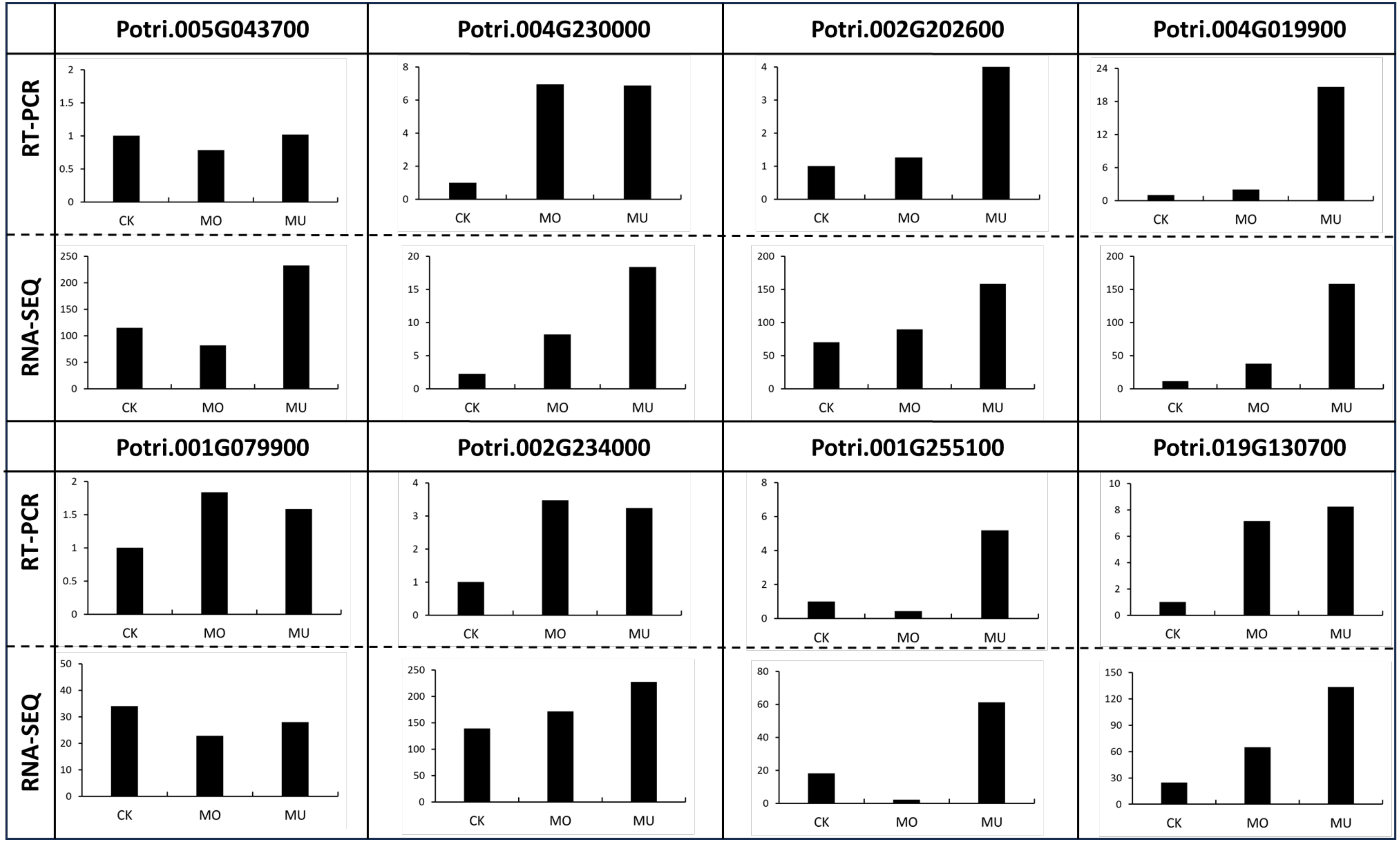

Supplementary Figure S3. Annotation and expression profiles of genes involved in plant-pathogen interaction (ko04626) enriched by the DEGs of MU_treat vs. control.

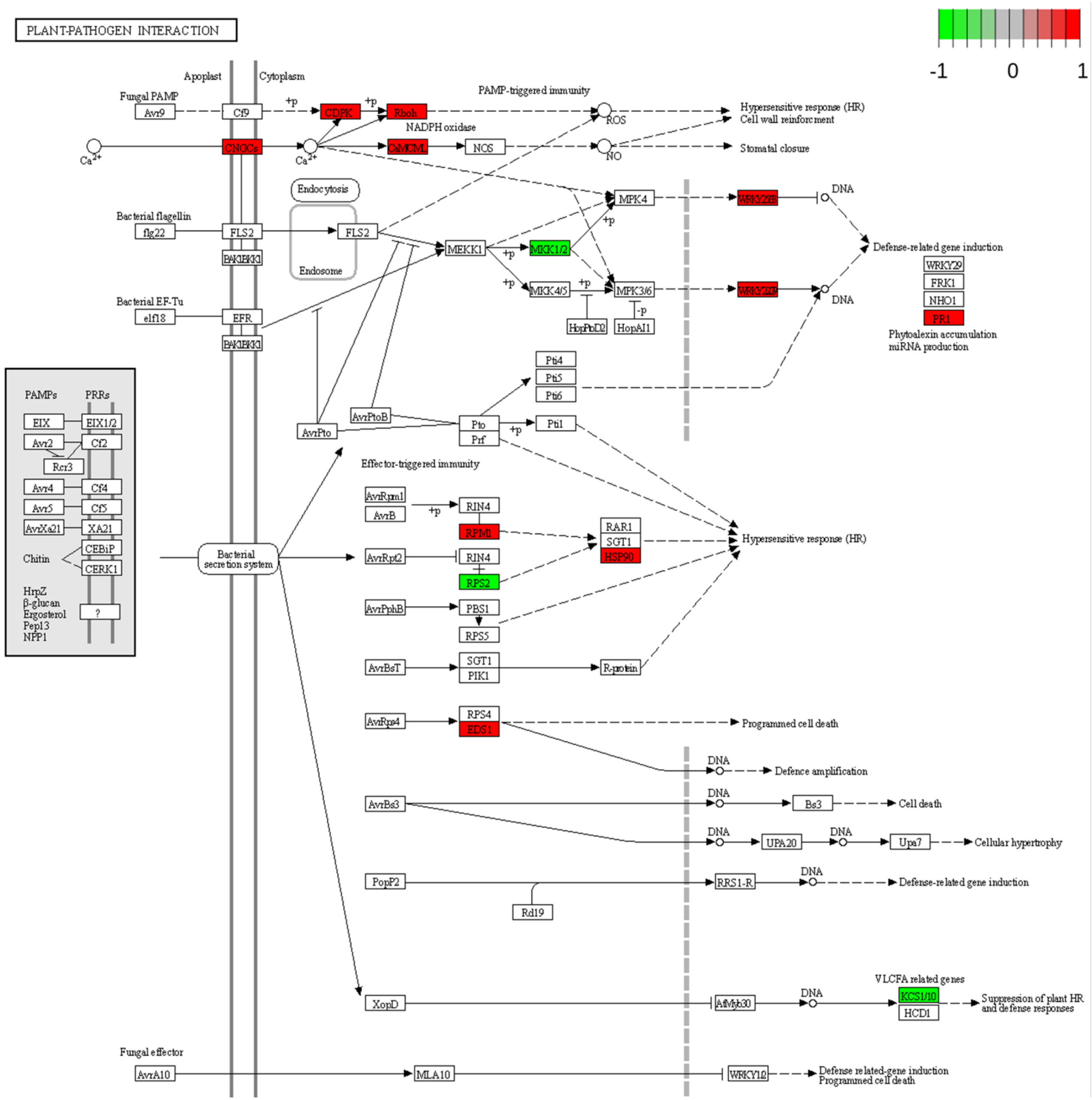

Potri.001G234500 :	3-ketoacyl-CoA synthase
Potri.002G178000 :	3-ketoacyl-CoA synthase
Potri.006G218000 :	3-ketoacyl-CoA synthase
Potri.009G116900 :	3-ketoacyl-CoA synthase
Potri.018G032200 :	3-ketoacyl-CoA synthase
Potri.012G023300 :	3-ketoacyl-CoA synthase
Potri.015G066200 :	3-ketoacyl-CoA synthase
Potri.001G134425 :	3-ketoacyl-CoA synthase
Potri.001G134701 :	3-ketoacyl-CoA synthase
Potri.009G083100 :	3-ketoacyl-CoA synthase
Potri.001G314900 :	calcium-binding protein CML
Potri.001G332900 :	calcium-binding protein CML
Potri.002G001400 :	calcium-binding protein CML
Potri.002G077300 :	calcium-binding protein CML
Potri.005G183300 :	calcium-binding protein CML
Potri.005G259900 :	calcium-binding protein CML
Potri.006G112500 :	calcium-binding protein CML
Potri.010G081000 :	calcium-binding protein CML
Potri.016G142000 :	calcium-binding protein CML
Potri.017G029700 :	calcium-binding protein CML
Potri.006G052900 :	calcium-dependent protein kinase
Potri.006G200600 :	calcium-dependent protein kinase
Potri.012G023300 :	calcium-dependent protein kinase
Potri.015G066200 :	calcium-dependent protein kinase
Potri.001G134425 :	calcium-dependent protein kinase
Potri.001G134701 :	calcium-dependent protein kinase
Potri.009G083100 :	calmodulin
Potri.010G146900 :	calmodulin
Potri.009G010700 :	cyclic nucleotide gated channel, plant
Potri.014G097900 :	cyclic nucleotide gated channel, plant
Potri.017G089800 :	cyclic nucleotide gated channel, plant
Potri.017G089900 :	cyclic nucleotide gated channel, plant
Potri.001G134300 :	disease resistance protein RPM1
Potri.001G134400 :	disease resistance protein RPM1
Potri.001G134425 :	disease resistance protein RPM1
Potri.001G134701 :	disease resistance protein RPM1
Potri.009G083100 :	disease resistance protein RPM1
Potri.001G134701 :	disease resistance protein RPM1
Potri.002G216800 :	disease resistance protein RPM1
Potri.018G013600 :	disease resistance protein RPM1
Potri.019G013140 :	disease resistance protein RPS2
Potri.015G069600 :	enhanced disease susceptibility 1 protein
Potri.005G241100 :	heat shock protein 90kDa beta
Potri.012G043200 :	mitogen-activated protein kinase kinase 1
Potri.009G082900 :	pathogenesis-related protein 1
Potri.009G083100 :	pathogenesis-related protein 1
Potri.009G083300 :	pathogenesis-related protein 1
Potri.001G098300 :	respiratory burst oxidase
Potri.003G133300 :	respiratory burst oxidase
Potri.003G133400 :	respiratory burst oxidase
Potri.003G159800 :	respiratory burst oxidase
Potri.006G137300 :	respiratory burst oxidase
Potri.015G109800 :	respiratory burst oxidase
Potri.001G099001 :	WRKY transcription factor 22
Potri.016G128300 :	WRKY transcription factor 33

Supplementary Figure S4. The expression level of genes belonged to the module "Peroxidases" in Mapman analysis.

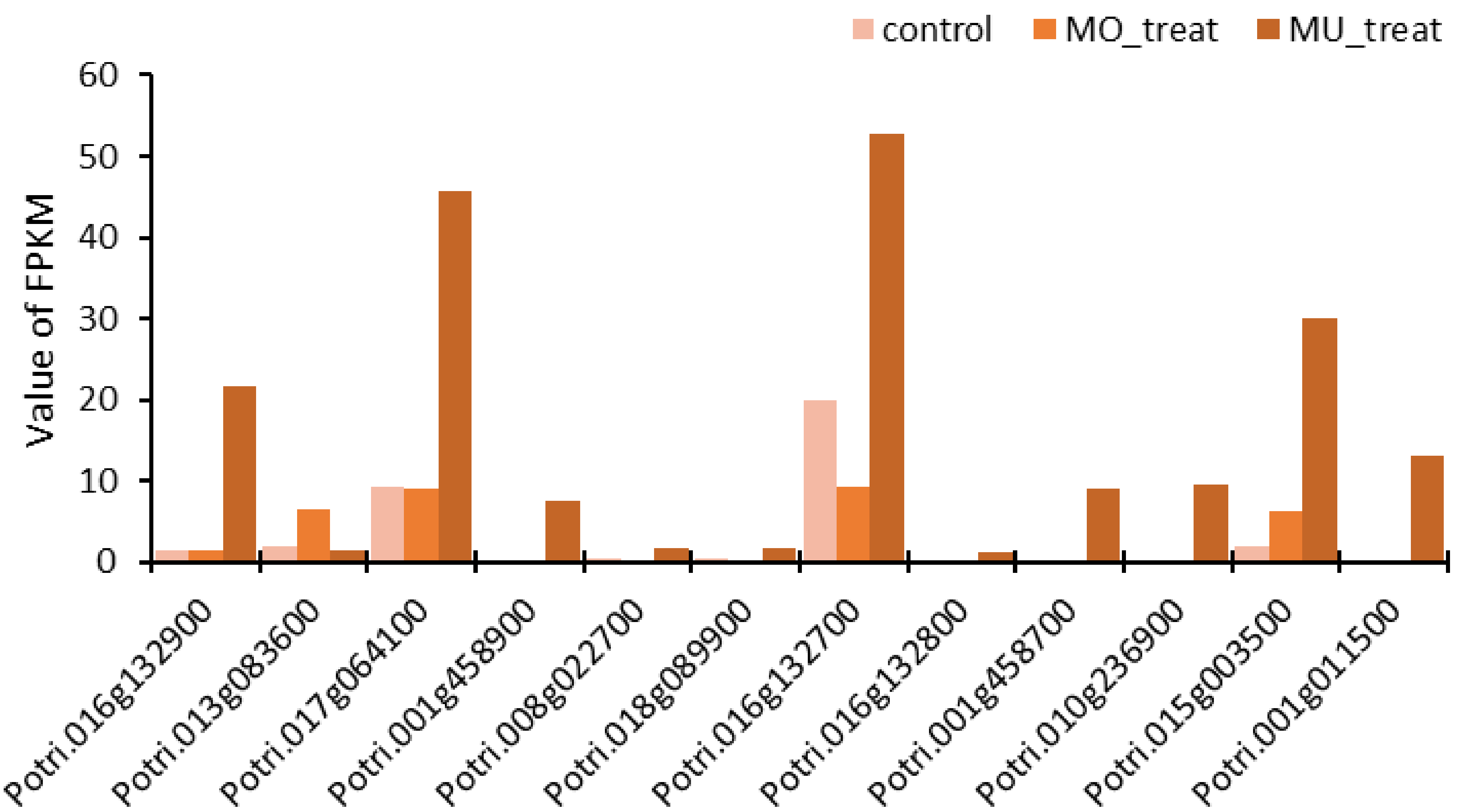

Supplementary Figure S5. The enriched GO terms (biological process) analyzed by the DEGs of MO_treat vs. MU_treat and NL895-H vs. NL895-W. (**A**) the GO terms enriched by DEGs of NL895-H vs. NL895-W, NL895 is a poplar clone containing high resistance to weak active strain of *Marssonina brunnea* f. sp. *multigermtubi* (W) and showed certain susceptible to high active strain (H) (Chen. C *et al*, 2015. "A comprehensive analysis of the transcriptomes of *Marssonina brunnea* and infected poplar leaves to capture vital events in host-pathogen interactions"). (**B**) the GO terms enriched by DEGs of MO_treat vs. MU_treat in this study.

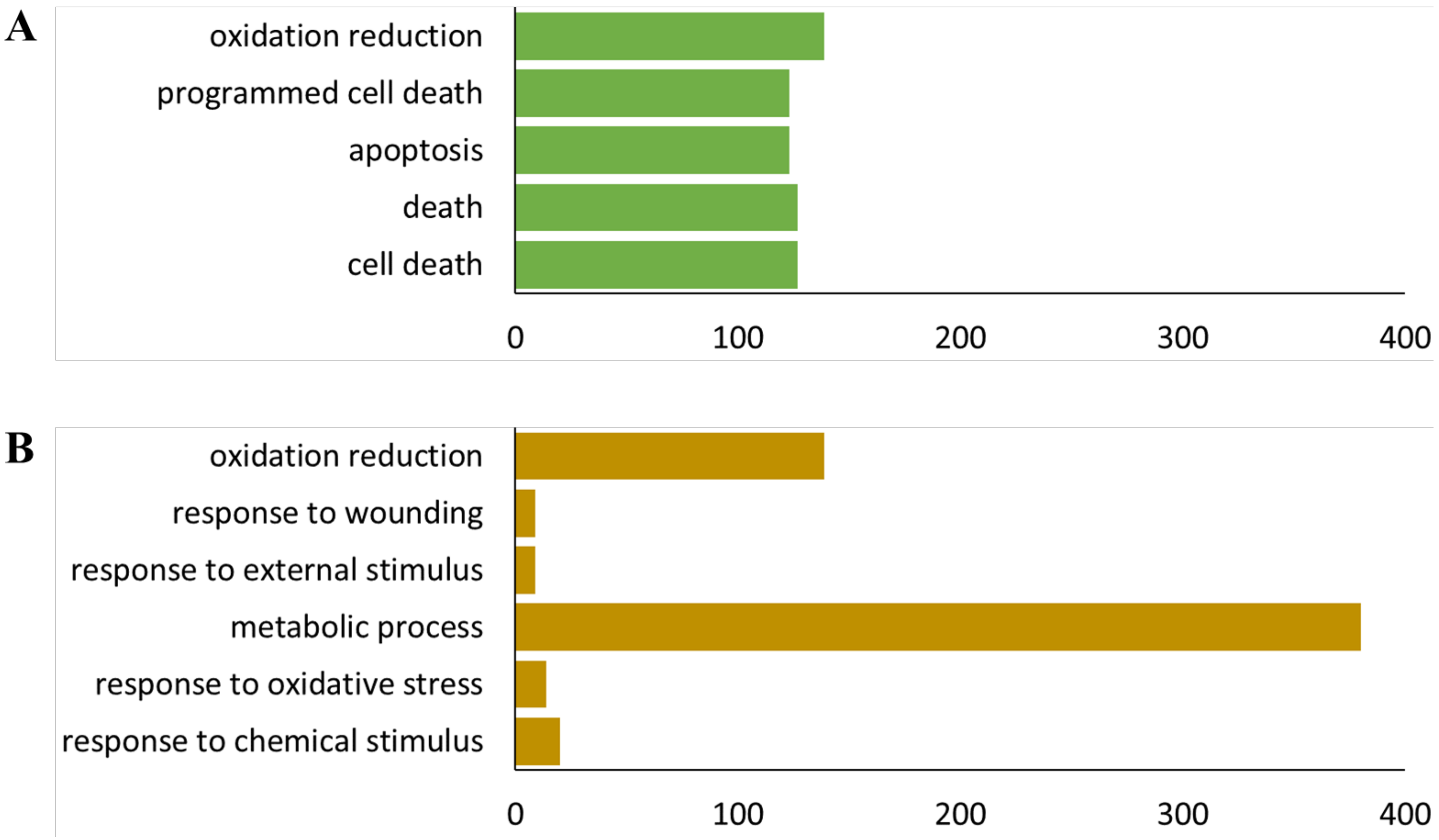

Supplement: Supplementary file 1 [file genes-15-00116-s001.zip › Supplementary Figures.pdf]
